# ATLAS: a Snakemake workflow for assembly, annotation, and genomic binning of metagenome sequence data

**DOI:** 10.1186/s12859-020-03585-4

**Published:** 2020-06-22

**Authors:** Silas Kieser, Joseph Brown, Evgeny M. Zdobnov, Mirko Trajkovski, Lee Ann McCue

**Affiliations:** 1 Department of Cell Physiology and Metabolism, Faculty of Medicine, Centre Medical Universitaire, 1206 Geneva, Switzerland; 2 Swiss Institute of Bioinformatics, Geneva, Switzerland; 3 Earth and Biological Sciences Directorate, Pacific Northwest National Laboratory, Richland, WA 99352 USA; 4 Current address: Department of Human Genetics, University of Utah, 15 S 2030 E, Salt Lake City, UT 84112 USA; 5 Institute of Genetics and Genomics in Geneva (iGE3), University of Geneva, 1206 Geneva, Switzerland; 6 Department of Genetic Medicine and Development, University of Geneva, 1206 Geneva, Switzerland; 7 Diabetes Center, Faculty of Medicine, Centre Medical Universitaire, 1206 Geneva, Switzerland

**Keywords:** Metagenomics, Analysis workflow, Annotation, Metagenome-assembled genomes

## Abstract

**Background:**

Metagenomics studies provide valuable insight into the composition and function of microbial populations from diverse environments; however, the data processing pipelines that rely on mapping reads to gene catalogs or genome databases for cultured strains yield results that underrepresent the genes and functional potential of uncultured microbes. Recent improvements in sequence assembly methods have eased the reliance on genome databases, thereby allowing the recovery of genomes from uncultured microbes. However, configuring these tools, linking them with advanced binning and annotation tools, and maintaining provenance of the processing continues to be challenging for researchers.

**Results:**

Here we present ATLAS, a software package for customizable data processing from raw sequence reads to functional and taxonomic annotations using state-of-the-art tools to assemble, annotate, quantify, and bin metagenome data. Abundance estimates at genome resolution are provided for each sample in a dataset. ATLAS is written in Python and the workflow implemented in Snakemake; it operates in a Linux environment, and is compatible with Python 3.5+ and Anaconda 3+ versions. The source code for ATLAS is freely available, distributed under a BSD-3 license.

**Conclusions:**

ATLAS provides a user-friendly, modular and customizable Snakemake workflow for metagenome data processing; it is easily installable with conda and maintained as open-source on GitHub at https://github.com/metagenome-atlas/atlas.

## Background

Metagenomics has transformed microbial ecology studies with the ability to generate genome sequence information from environmental samples, yielding valuable insight into the composition and functional potential of natural microbial populations from diverse environments [1, 2]. Despite the prevalence of metagenome data, there are few broadly accepted standard methods, either for the generation of that data [3–5] or for its processing [6, 7]. In particular, processing metagenome data in an efficient and reproducible manner is challenging because it requires implementation of several distinct tools, each designed for a specific task.

The most direct and frequently used way to analyze metagenome data is to map the sequence reads to reference genomes, when a suitable genome database from cultivated microbes is available (e.g. Humann2 [8]). However, these methods do not capture uncultivated species; studies using single-copy phylogenetic marker genes have improved estimates of species richness in metagenome data by expanding the representation of uncultivated species [9]. To truly characterize a natural microbial community and examine its functional potential, assembly-based metagenome analyses are needed. This has been demonstrated by recent studies that have recovered thousands of new genomes using co-abundance patterns among samples to bin contigs into clusters [10–13].

A number of assembly-based metagenome pipelines have been developed, each providing a subset of the required tools needed to carry out a complete analysis process from raw data to annotated genomes [14–17]. For example, MOCAT2 [16] relies on gene catalogs to evaluate the functional potential of the metagenome as a whole, but without directly relating functions to individual microbes. Metagenome processing pipelines commonly default to co-assembly of the samples rather than assembly of individual samples, resulting in more fragmented assemblies [18]. Only some applications (e.g., IMP [17]) permit the co-assembly of metagenomes and metatranscriptomes for individual samples. Furthermore, the configuration and technical constraints to user control often limit the adoption of these tools in the research community.

Here we present an entirely new version of ATLAS [19], an assembly-based pipeline for the recovery of genes and genomes from metagenomes, that produces annotated and quantified genomes from multiple samples in one run with as little as three commands. The pipeline integrates state-of-the art tools for quality control, assembly and binning. The installation of ATLAS is automated: it depends only on the availability of Anaconda and installs all dependencies and databases on the fly. The internal use of Snakemake [20] allows efficient and automated deployment on a computing cluster.

### Implementation

**Fig. 1:**
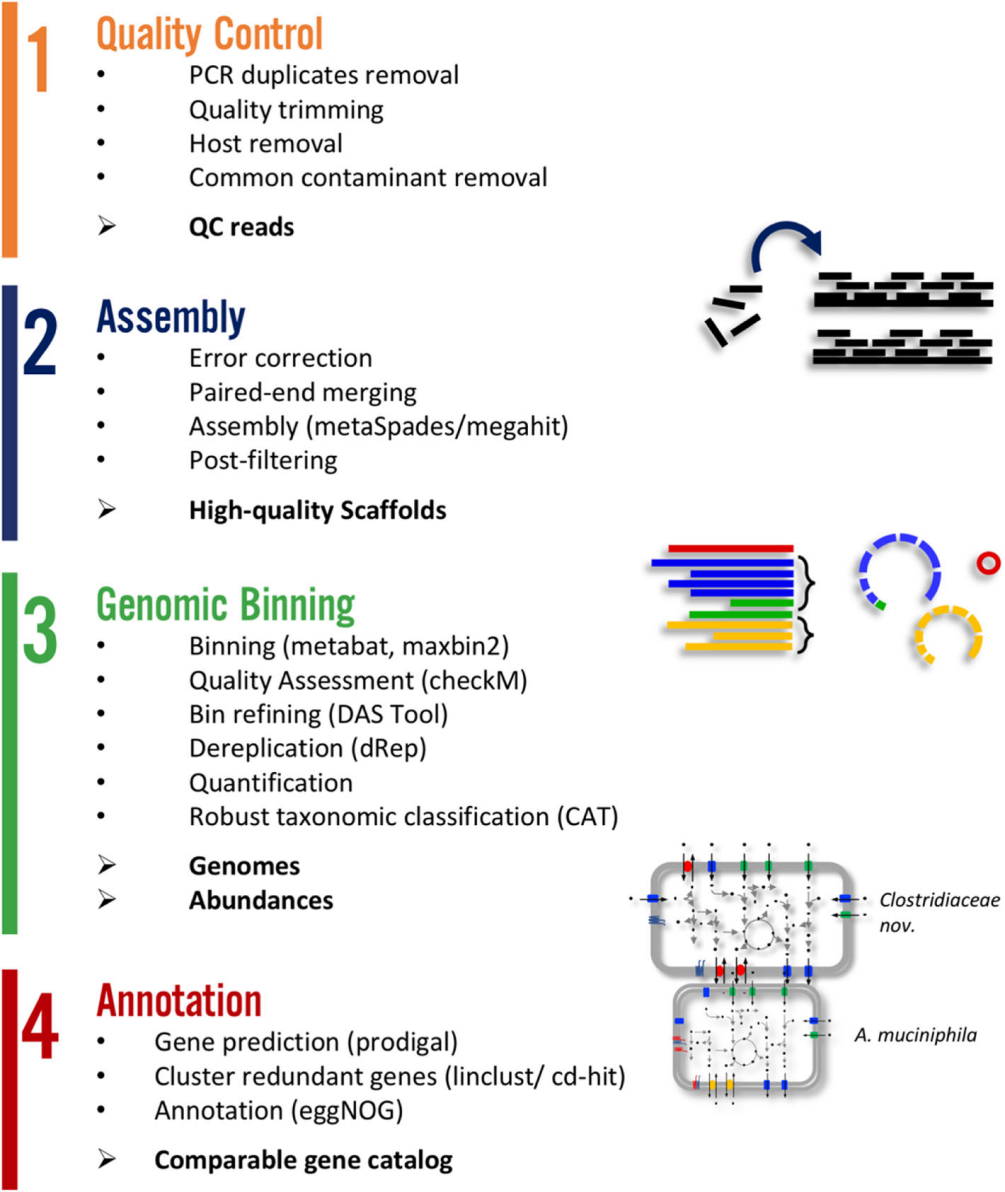
The ATLAS workflow. This high-level overview of the protocol captures the primary goal of the sub-commands that can be executed by the workflow. Individual modules can be accessed via the command line or the entire protocol can be run starting from raw sequence data in the form of single- or paired-end FASTQ files

The ATLAS framework organizes sequence data processing tools into four distinct analysis modules: [1] quality control, [2] assembly, [3] genome binning and [4] annotation (Fig. 1); each module can be run independently, or all four modules combined in a complete analysis workflow. ATLAS is implemented in Python and uses the Snakemake [20] workflow manager for extensive control of external tools, including versioning of configurations and environments, provenance capabilities, and scalability on high-performance computing clusters. ATLAS uses Anaconda [21] to simplify initial deployment and environment set-up, and dependencies are handled by Bioconda [22] at runtime. Complete usage and user options are outlined in the ATLAS documentation (https://metagenome-atlas.rtfd.io).

#### Quality control

Quality control of raw sequence data, in the form of single- or paired-end FASTQ files, is performed using utilities in the BBTools suite [23]. Specifically, *clumpify* is used remove PCR duplicates and compress the raw data files, followed by *BBduk* to remove known adapters, trim and filter reads based on their quality and length (respectively), and error-correct overlapping paired-end reads where applicable. *BBSplit* is used to remove contaminating reads using reference sequences: PhiX is provided as a default or can be replaced by user-specified fasta-format sequences. To optimize data use, reads that lose their mate during these steps are seamlessly integrated into the later steps of the pipeline.

#### Assembly

Prior to metagenome assembly, ATLAS uses additional BBTools utilities [23] to perform an efficient error correction based on k-mer coverage (*Tadpole*) and paired-end read merging (*bbmerge*). If paired-end reads do not overlap, *bbmerge* can extend them using read-derived overlapping k-mers. ATLAS uses metaSPAdes [24] or MEGAHIT [25, 26] for de novo assembly, with the ability to control parameters such as k-mer lengths and k-mer step size for each assembler, as well as hybrid-assembly of paired short- and long-read libraries. The quality-controlled reads are mapped to the assembled contigs, and bam files are generated to facilitate downstream calculations that may be of interest (e.g., calculating contig coverage). The assembled contigs shorter than a minimal length, or without mapped reads, are filtered out to yield high-quality contigs.

#### Genome binning

The prediction of metagenome-assembled genomes (MAGs) allows organism-specific analyses of metagenome datasets. In ATLAS, two binning methods are implemented (Fig. 1): metabat2 [27] and maxbin2 [28]. These methods use tetra-nucleotide frequencies, differential abundance, and/or the presence of marker genes as criteria. ATLAS supports assembly and binning for each sample individually, which produces more continuous genomes than co-assembly [29]. Definition of which samples are likely to contain the same bacterial species, via a group attribute in the Snakemake configuration file, supports binning based on co-abundance patterns across samples. Reads from all of the samples defined in a group are then aligned to the individual sample assemblies, to obtain the co-abundance patterns needed for efficient binning. The bins produced by the different binning tools can be combined using the dereplicate, aggregate and score tool (DAS Tool, [30]), to yield MAGs for each sample. Finally, the completeness and contamination of each MAG are assessed using CheckM [31].

Because the same genome may be identified in multiple samples, dRep [29] is used to obtain a non-redundant set of MAGs for the combined dataset by clustering genomes to a defined average nucleotide identity (ANI, default 0.95) and returning the representative with the highest dRep score in each cluster. dRep first filters genomes based on genome size (default > 5000 bp) and quality (default > 50% completeness, < 10% contamination), then clusters the genomes using Mash [32], followed by MUMmer [33], thereby benefitting from their combined speed (Mash) and accuracy (MUMmer). The abundance of each genome can then be quantified across samples by mapping the reads to the non-redundant MAGs and determining the median coverage across each the genome.

#### Taxonomic and functional annotation

For annotation, ATLAS supports the prediction of open reading frames (ORFs) using Prodigal [34]. The translated gene products are then clustered using linclust [35] or mmseqs [36] to generate non-redundant gene and protein catalogs, which are mapped to the eggNOG catalogue v5 [37, 38]. Robust taxonomic annotation is performed using the genome taxonomy database tool kit (GTDB-tk, [39]). In addition, phylogenetic trees are built based on the markers from GTDB and CheckM.

#### Output

The ATLAS output for each sample includes the quality-controlled reads, assembled contigs, bam files (reads mapped to contigs), and predicted genome bins, together with summary statistics in an HTML report. The final output includes results from all samples, including the raw and normalized counts for the set of non-redundant, high-quality MAGs, with a quality report and their inferred taxonomy. From the annotation stage, two fasta files are produced containing the nucleotide and amino acid sequences of the representative genes in the non-redundant gene catalog, together with a table containing the gene annotations summarized at the genome level.

**Fig. 2:**
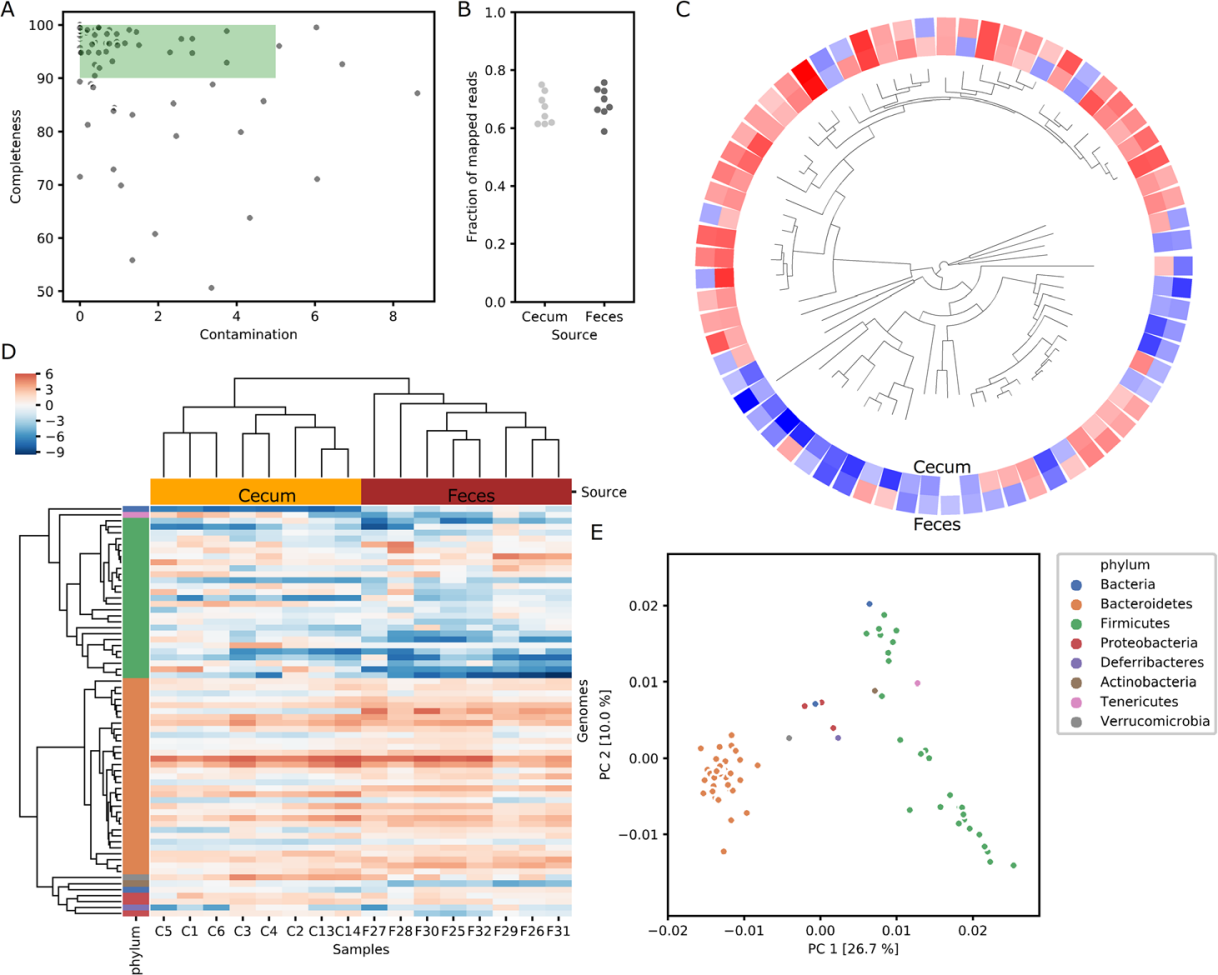
Example output from the ATLAS workflow. Fecal microbiome data (PRJNA480387 [40];) processed by ATLAS show: A) the completeness and contamination of dereplicated MAGs, with high-quality genomes highlighted; B) the fraction of reads mapped to genomes; C) a phylogenetic tree of MAGs with average abundance in feces and cecum on a centered log_2_ scale; D) a heatmap of abundance on a centered log_2_ scale in which MAGs were clustered by phylogenetic distance and samples by Euclidian distance; E) a principle components analysis of the MAGs based on functional annotation

Figure 2 shows examples of ATLAS output in which we analyzed the metagenome data from paired feces and cecum samples of 8 mice fed ad libitum (PRJNA480387 [40];). On average, the sample data contained 3.5 Gbp, and produced assemblies of 108 Mbp per sample. There were 374 MAGS predicted (completeness > 50% and contamination < 10%), that formed 69 non-redundant clusters (ANI > 99%; Fig. 2A). These genomes account for 75% of the reads (Fig. 2B). In general, *Bacteroides* were more abundant than *Firmicutes*, in both cecum and feces (Fig. 2C,D). A principal coordinates analysis based on the functional annotation revealed two functionally distinct clusters of Firmicutes (Fig. 2E). Details of these results are provided on GitHub (https://github.com/metagenome-atlas/supp_data_atlas).

## Conclusions

ATLAS is easy to install and provides documented and modular workflows for the analysis of metagenome data. The internal codes utilized by the workflow are highly configurable using either a configuration file or via the command line. ATLAS provides a robust bioinformatics framework for high-throughput sequence data, where raw FASTQ files can be fully processed into annotated tabular files for downstream analysis and visualization. ATLAS fills a major analysis gap, namely the integration of tools for quality control, assembly, binning and annotation, in a manner that supports robust and reproducible analyses. ATLAS provides these analysis tools in a command-line interface amenable to high-performance computing clusters.

The source code for ATLAS is distributed under a BSD-3 license and is freely available at https://github.com/metagenome-atlas/atlas, with example data provided for testing. Software documentation is available at https://metagenome-atlas.rtfd.io, which describes the installation and use of ATLAS including a Docker container (https://hub.docker.com/r/metagenomeatlas/atlas).

**Availability** Project name: ATLAS.

Project home page: https://github.com/metagenome-atlas/atlas

Archived version: 10.1101/737528

Operating system(s): Linux.

Programming language: Snakemake/Python.

Other requirements: Miniconda.

License: BSD-3.

Any restrictions to use by non-academics: None.

## Data Availability

Not applicable.
